# SARS-CoV-2 pseudoparticles preferentially infect ectoderm in human embryonic tissues

**DOI:** 10.3389/fcell.2026.1733662

**Published:** 2026-04-07

**Authors:** Ann Song, Prue Talbot

**Affiliations:** 1 Cell, Molecular, and Developmental Biology Graduate Program, University of California, Riverside, CA, United States; 2 Department of Molecular, Cell, and Systems Biology, University of California, Riverside, CA, United States

**Keywords:** COVID-19, disease-in-a-dish, ectoderm, embryonic stem cells, SARS-CoV-2, teratogens, tropism

## Abstract

**Introduction:**

The early stages of human embryonic development are challenging to study in pregnant women.

**Methods:**

A “disease-in-a-dish” model was utilized to investigate SARS-CoV-2 infection of human embryonic stem cells and the three germ layers (ectoderm, endoderm, and mesoderm).

**Results:**

Ectodermal cells showed significantly higher infection rates compared to the other cell types. This increased susceptibility was attributed to three key factors characteristic of the ectoderm: dual viral entry pathways (membrane fusion and endocytosis), elevated TMPRSS2 activity, and a markedly reduced glycocalyx, which facilitated viral access to host cell receptors.

**Discussion:**

Our findings provide strong evidence that cells in early post-implantation human embryos are susceptible to SARS-CoV-2 infection. The high level of infection in the ectodermal cells raises concern for potential teratogenic effects, particularly involving the nervous system. Future clinical studies should investigate neurological outcomes in infants born to mothers infected with SARS-CoV-2 during pregnancy.

## 1 Introduction

During the COVID-19 pandemic, numerous studies were conducted on human-to-human viral transmission (Guo et al., 2020; Rahman et al., 2020), as well as on vaccine (Jackson et al., 2020; Killeen et al., 2023), and therapeutic development (Arbel et al., 2022; Akinosoglou et al., 2022). In contrast, relatively few reports have examined vertical transmission of SARS-CoV-2 from mother to embryo or fetus in pregnant women with COVID-19 (Kotlyar et al., 2021). The first documented case of vertical transmission occurred in a pregnant woman infected during her third trimester (Vivanti et al., 2020). Maternal SARS-CoV-2 infection has been associated with adverse reproductive outcomes (Young et al., 2022), including an increased risk of pre-eclampsia (Mendoza et al., 2020; Villar et al., 2021; Gurol-Urganci et al., 2021; Wei et al., 2021), premature rupture of the membranes (Allotey et al., 2020), Cesarean delivery (Metz et al., 2021; Gurol-Urganci et al., 2021), and miscarriage (Gurol-Urganci et al., 2021). While adverse outcomes such as preterm birth and low birth weight are frequently observed in these pregnancies (Metz et al., 2021; Villar et al., 2021), it is difficult to distinguish direct viral effects on the fetus from secondary consequences of maternal illness, such as systemic inflammation and hypoxia.

*In utero* transmission has been well documented during the late third trimester, with evidence of transplacental passage of SARS-CoV-2 based on qPCR and immunohistochemistry of amniotic fluid, cord blood, and placental tissues (Vivanti et al., 2020; Hosier et al., 2020). The strongest evidence of early gestational transmission comes from a study of a stillborn first trimester fetus, in which SARS-CoV-2 was detected in placental and fetal tissues by qPCR, immunofluorescence microscopy, and electron microscopy (Valdespino-Vázquez et al., 2021). Viral presence in fetal lungs and kidneys was associated with inflammation and organ damage. Similarly, Fenizia et al. (2022) reported that 30% of embryos/fetuses and 20% of syncytiotrophoblasts from first-trimester pregnancies voluntarily terminated by SARS-CoV-2–positive women tested positive for the virus, using qPCR and QuantiGene assays. Estimates of the rate of transmission vary significantly across studies, likely due to small sample sizes and differences in diagnostic timing. For instance, Kotlyar et al. (2021) reported a vertical transmission rate of approximately 3.2% based on a meta-analysis, whereas Fenizia et al. (2022) detected the virus in 30% of embryos/fetuses from first-trimester pregnancies. These findings confirm that vertical transmission can occur, although additional large-scale studies are required to accurately determine the rate of transmission from infected mothers to their embryos/fetuses throughout gestation.

The infection potential of human embryos and fetuses by SARS-CoV-2 raises concerns regarding embryonic lethality and teratogenic outcomes. Several viruses are established teratogens, such as Zika virus, which causes microcephaly (Brasil et al., 2016) and cytomegalovirus (CMV), which can cause deafness (Carlson et al., 2010). Emerging evidence suggests that SARS-CoV-2 may also exert teratogenic effects in humans, such as neurodevelopmental delays and altered cortical brain structure (Edlow et al., 2022; Peng et al., 2025). Clinical studies of pregnancies affected by maternal COVID-19 have documented a spectrum of adverse fetal outcomes, including restricted fetal growth (Gulersen et al., 2020; Bui et al., 2024), decreased fetal movement (Favre et al., 2021), fetal distress (Villar et al., 2021; Wei et al., 2021; Bui et al., 2024; Zia et al., 2024), stillbirths (Gurol-Urganci et al., 2021; Allotey et al., 2020; Li A. et al., 2024), preterm births (Chinn et al., 2021; Metz et al., 2021; Gurol-Urganci et al., 2021; Villar et al., 2021; Wei et al., 2021; Li J. et al., 2024; Li A. et al., 2024; Zia et al., 2024), neonatal mortality (Villar et al., 2021; Bui et al., 2024), admission to neonatal intensive care units (Metz et al., 2021; Villar et al., 2021; Li A. et al., 2024), and low birth weight (Metz et al., 2021; Villar et al., 2021; Wei et al., 2021; Li A. et al., 2024). Importantly, some developmental anomalies linked to SARS-CoV-2 exposure may not manifest until later in life, analogous to the delayed onset of vaginal cancer following *in utero* exposure to diethylstilbestrol (Veurink et al., 2005). These findings emphasize the need for continued investigation into the teratogenic potential of SARS-CoV-2 and its long-term consequences for exposed offspring.

While first- and third-trimester studies provide robust evidence of vertical transmission, the susceptibility of embryonic tissues during the earliest stages of development remains poorly understood. To address this knowledge gap, we used a “disease-in-a-dish-model” based on SARS-CoV-2 pseudoparticles to study viral entry into 4 cell types representative of weeks 1-4 of human prenatal development (Song et al., 2023a; Song et al., 2023b). Given the ethical constraints surrounding experimentation during early pregnancy (Lin and Talbot, 2011), this *in vitro* approach offers a tractable alternative for mechanistic study.

Human embryonic stem cells (hESCs), which have the properties of epiblast cells in post-implantation embryos (Nichols and Smith, 2009; 2011), were examined in conjunction with cells of the three germ layers. SARS-CoV-2 entry requires host cell expression of the ACE2 receptor and TMPRSS2 protease: spike protein binding to ACE2 at the plasma membrane is followed by TMPRSS2 mediated cleavage at the S2 site, facilitating membrane fusion (Zeng et al., 2022; Colaco et al., 2021; Weatherbee et al., 2020; Hoffmann et al., 2020; Cai et al., 2020). In the absence of surface TMPRSS2 activity, spike-ACE2 binding may instead trigger endocytosis, with subsequent activation by endosomal proteases such as cathepsin L—thus enabling viral entry via either fusion or endocytosis (Supplementary Figure S1).

The pseudoparticle system incorporates a fluorescent viral backbone, allowing real-time visualization of viral uptake and quantification via flow cytometry (Supplementary Figure S2). This platform enabled us to delineate the entry mechanisms of SARS-CoV-2 in hESCs and the three germ layers, identify pharmacological inhibitors of infection, and elucidate the molecular basis of tissue tropism during early human development.

## 2 Materials and methods

### 2.1 Tissue culture and reagents

H9 hESCs (WA09, female, hPSCreg: WAe009-A (ID/registry); Passage 27) were purchased from WiCell (Madison, WI). Upon thawing, cells were passaged no more than 10 times for experimental seeding. G-T-L banding karyotype, short tandem repeat analysis, *Mycoplasma* testing, and sterility testing were performed by WiCell.

H9 hESCs were grown in complete mTeSR plus culture medium (STEMCELL Technologies, Vancouver, Canada) (Lin and Talbot, 2011; Song et al., 2023a). Cells were maintained on Matrigel-coated 6-well plates until they reached approximately 80% confluency. For passaging, cells were gently washed once with phosphate-buffered saline (PBS) and removed from the well with 1 mL of ReLeSR (STEMCELL Technologies, Vancouver, Canada). ReLeSR was immediately removed, and colonies were incubated at 37 °C for 4 min, then 1 mL of mTeSR plus was gently added to the well to collect undifferentiated cells. A 1:10 split ratio was used to passage to a new 6-well plate. Cryostor 10 (STEMCELL Technologies, Vancouver, Canada) was used for freezing, and cells were thawed in mTeSR plus medium based on the manufacturer’s recommendation.

HEK 293T and an ACE2-overexpressing cell line (HEK 293T-ACE2) (ATCC, Manassas, VA) were grown in DMEM with high glucose and 10% FBS (Gibco, Carlsbad, CA). Both cell types were grown in T25 flasks until they reached approximately 90% confluency. For passaging, cells were washed once with PBS, lifted from the flask with 1.25 mL of 0.25% trypsin-EDTA for 3 min at 37 °C, and then centrifuged to remove the supernatant. The pellet was resuspended in complete growth medium.

The following small molecule inhibitors were used to target TMPRSS2: ambroxol (10 μM; TCI Chemicals, Portland, OR), aprotinin (10 μM; Tocris, San Diego, CA), Camostat (10 μM; Sigma-Aldrich, Burlington, MA), Nafamostat (10 μM; TCI Chemicals, Portland, OR). Endocytosis inhibitors that were used included: Dyngo4a (20 μM, Selleckchem, Houston, TX), Pitstop2 (20 μM; Abcam, Cambridge, MA), OcTMAB (10 μM; Abcam, Cambridge, MA), MiTMAB (10 μM; Abcam, Cambridge, MA), mβCD (20 μM; Sigma-Aldrich, Burlington, MA), Nystatin (20 μM; Sigma-Aldrich, Burlington, MA), and Filipin (20 μM; Sigma-Aldrich, Burlington, MA).

### 2.2 Trilineage differentiation

To simulate early embryonic development, H9 hESCs were differentiated into the three germ layers using the STEMdiff™ trilineage differentiation kit (STEMCELL Technologies, Vancouver, Canada) according to the manufacturer’s protocol. Single cells of H9 were obtained after treatment with gentle cell dissociation reagent (STEMCELL Technologies, Vancouver, Canada). Cells were plated on Matrigel-coated 48-well plates in mTeSR plus medium with ROCK inhibitor (10 μM; Tocris, San Diego, CA): 1 × 10^5^ cells per well for ectoderm and endoderm and 2.5 × 10^4^ for mesoderm and the undifferentiated control. Every 24 h a medium change of each differentiation medium was performed. Samples were infected with SARS-CoV-2 pseudoparticles after 5 days (mesoderm and endoderm) or 7 days (ectoderm and undifferentiated H9) of seeding.

### 2.3 Immunofluorescence microscopy

The cells were seeded in 8-well chamber slides (Ibidi; Gräfelfing, Germany). After differentiation, the cells were fixed at room temperature in 4% paraformaldehyde in PBS for 15 min and washed with PBS. For ACE2 and TMPRSS2 labeling, cells were treated with 50 mM DTT and 6 M guanidine-HCl for 5 min prior to quenching with 100 mM iodoacetamide. For other antibodies, cells were permeabilized with 0.1% Triton X-100 for 10 min. The cells were then blocked with 10% donkey serum. Primary antibodies were diluted in blocking buffer. The following primary antibodies were used: mouse anti-PAX6 (1:60; Developmental Studies Hybridoma Bank, Iowa City, IA), goat anti-SOX17 (1:200; R&D Systems, Minneapolis, MN), mouse anti-NCAM (1:30; Developmental Studies Hybridoma Bank, Iowa City, IA), goat anti-ACE2 (1:200; R&D Systems, Minneapolis, MN), and mouse anti-TMPRSS2 (1:200; Santa Cruz Biotechnology, Dallas, TX).

Following overnight incubation with primary antibody at 4 °C, the cells were washed three times with 0.2% Tween in PBS. Next, the cells were incubated in the dark with Alexa Fluor-conjugated fluorescent secondary antibodies (1:500; Invitrogen, Carlsbad, CA). After washing in PBS, the cells were mounted with diluted Vectashield and DAPI for nuclear staining. A Nikon Eclipse Ti inverted microscope was used to image at 40x. The NIS Elements Software and ImageJ software were used to image and analyze the data.

To establish the location of surface proteins (ACE2, TMPRSS2), fixed cells were labeled with the antibody and fluorescence lectin conjugates (WGA-FITC or Con A-FITC; 1:100, Vector Laboratories, Newton, CA, USA). Antibody staining, mounting, and imaging steps were performed as above. To determine surface glycosylation patterns in the 4 cell types (hESCs, endoderm, mesoderm, and ectoderm), cells were labeled with various lectins (Con A, DBA, PNA, RCA120, SBA, UEA I, WGA; Vector Laboratories, Newton, CA, USA).

### 2.4 TMPRSS2 activity assay

TMPRSS2 fluorogenic substrate, Boc-Gln-Ala-Arg-AMC HCl (2.5 mM, Bachem), was prepared in 50 mM Tris (pH 8) and 150 mM NaCl. After differentiation, H9 hESCs and the differentiated germ layer cells were washed twice with PBS and lysed for 1 min on ice in RIPA buffer. The cells were then sheared with a 21-gauge needle, followed by centrifugation at 3,000 rpm for 5 min at 4 °C. The lysate protein was quantified using the Pierce BCA assay kit (Thermo Scientific, Waltham, MA). 10 μg of protein was added to each reaction well. The fluorogenic substrate was added to each well at a final concentration of 10 µM. Fluorescence intensity was measured at 340/440 nm using a BioTek Synergy HTX, multi-mode microplate reader (Winooski, VT). To validate the inhibitory effects of aprotinin in ectoderm, ectodermal cells were preincubated with aprotinin for 24 h before lysate collection.

### 2.5 Lentiviral production to generate SARS-CoV-2 pseudoparticles

SARS-CoV-2 pseudoparticles were generated as described in our previous work (Song et al., 2023a; Song et al., 2023b). HEK293T cells were plated with antibiotic-free medium at a density of 7 × 10^6^ cells in a T75 flask and transfected using lentiviral plasmids (Song et al., 2023a; Song et al., 2023b) and a Lipofectamine3000 Kit (Invitrogen, Carlsbad, CA) according to the manufacturer’s protocol. After overnight incubation, fresh medium was added to cells supplemented with 1% BSA. Conditioned medium was collected and centrifuged 48 h post-transfection. The supernatant was filtered using a 0.45 µm Acrodisc syringe filter (Cytivia Life Sciences, Marlborough, MA), and the filtrate was mixed with 5x polyethylene glycol (Abcam, Cambridge, MA) and precipitated overnight at 4 °C. The lentivirus was collected by centrifugation, and the pellet was resuspended in Viral Re-suspension Solution (Abcam, Cambridge, MA). Virus aliquots were stored at −80 °C. Prior to use in experiments, the transduction efficiency of each batch of viruses was tested in HEK 293T-ACE2.

### 2.6 Pseudotyping of human SARS-CoV-2 pseudoparticles

H9 hESCs and the differentiated germ layers were infected with SARS-CoV-2 pseudoparticles using a multiplicity of infection (MOI) of 0.1. The medium was replaced after overnight incubation of the infected cells. Cells were dissociated with gentle cell dissociation reagent 48 h post-infection and washed three times with 0.5% BSA in PBS. After the final wash, the cells were resuspended in the same wash buffer and analyzed using flow cytometry. A Novocyte flow cytometer (Agilent Technologies, Santa Clara, CA) was used to detect ZsGreen in the FITC channel. The resulting flow cytometer files were analyzed using NovoExpress software. Mock infection was used as a background control. Prior to infection, the cells were preincubated for 2 h with TMPRSS2 inhibitors or endocytosis inhibitors, which were kept in the medium until harvesting for flow cytometry.

### 2.7 Endocytosis assay

Trilineage differentiation was performed in 8-well chamber slides. The cells were incubated overnight with TRITC-conjugated dextran (Invitrogen, Carlsbad, CA) with and without endocytosis inhibitors. After fixation in 4% paraformaldehyde, mounting was performed using diluted Vectashield with DAPI and images were collected using a Nikon Eclipse inverted microscope.

### 2.8 RNA extraction and quantitative real-time PCR analysis

RNA was extracted from hESCs and the germ layer cells using the RNeasy Mini Kit (Qiagen, Germantown, MD) according to the manufacturer’s protocol. RT-PCR was performed after generating first-strand cDNAs from 1 µg of total RNA using iScript Reverse Transcription Supermix (Bio-Rad, Hercules, CA), as recommended by the manufacturer. qPCR was performed using the SsoAdvanced Universal SYBR Green Supermix in a BIO-RAD CFX connect cycler (Bio-Rad Laboratories, Hercules, CA). Primer sequences are listed in Supplementary Table S1. Relative expression analysis is presented as 2^−ΔΔCt^ values normalized to the expression of ACTIN and relative to untreated (negative control) samples. All reactions were performed in triplicates.

### 2.9 Deglycosylation assay

hESCs, endoderm, and mesoderm were seeded in 48-well plates. After 24 h, the cells were treated with 0.006 U/mL or 0.018 U/mL of neuraminidase (Sigma-Aldrich, Burlington, MA) in DMEM at 37 °C for variable periods of time (0 min, 45 min, 90 min, 180 min, 360 min). 1 Unit of neuraminidase is defined to liberate 1.0 µM of N-acetyl neuraminic acid per minute at pH 5.0 at 37 °C using bovine submaxillary mucin. After deglycosylation, neuraminidase was quenched with culture medium and cells were washed with PBS.

For microscopy, cells were fixed in 4% paraformaldehyde for 15 min at room temperature, followed by WGA-FITC labeling and mounting with Vectashield containing DAPI. For infection, cells were transferred to their respective culture media, and SARS-CoV-2 pseudoparticles were added after the glycosidase treatments to allow infection. After 48 h, cells were collected and analyzed using flow cytometry.

### 2.10 Data analysis and statistics

For infection and TMPRSS2 activity data, the mean and standard error of the mean for three independent experiments were plotted using GraphPad Prism 10 software (GraphPad, San Diego, CA). For infection data, the mean of the DMSO group was set to 100, and the inhibitor groups were compared to this value. Statistical significance was determined using Minitab Statistics Software (Minitab, State College, PA). When the data were not normally distributed, they were subjected to a Box-Cox or logarithmic transformation, and the data were retested to confirm that they satisfied the analysis of variance (ANOVA) model (normal distribution and homogeneity of variances). Infection analyses were performed using a one-way ANOVA, while TMPRSS2 cleavage analysis was performed using a two-way ANOVA, in which the factors were time and cell type. When the means were significant (p < 0.05), groups treated with inhibitors were compared to each other using Tukey’s *post hoc* analysis or compared to the control group using Dunnett’s *post hoc* analysis. To analyze qPCR data, the Kruskal–Wallis nonparametric test was performed followed by Dunn’s *post hoc* test since the untreated control had no variance. For deglycosylation data at two concentrations of neuraminidase, unpaired two-tailed t-tests were performed using GraphPad Prism 10 software (GraphPad, San Diego, CA) to compare the infection levels against each other.

## 3 Results

### 3.1 The machinery for SARS-CoV-2 entry (ACE2 and TMPRSS2) is present on hESCs and germ layer cells

Directed differentiation of H9 hESCs into ectoderm, endoderm, and mesoderm was performed with the STEMdiff Trilineage Kit. Each germ layer was labeled with a lineage specific marker. None of the germ layer markers were expressed in the undifferentiated hESCs (Supplementary Figure S3). After differentiation, markers were expressed in the correct germ layer (endoderm: SOX17, mesoderm: NCAM, and ectoderm: PAX6) without cross reaction to other germ layers (Supplementary Figure S3).

**FIGURE 1:**
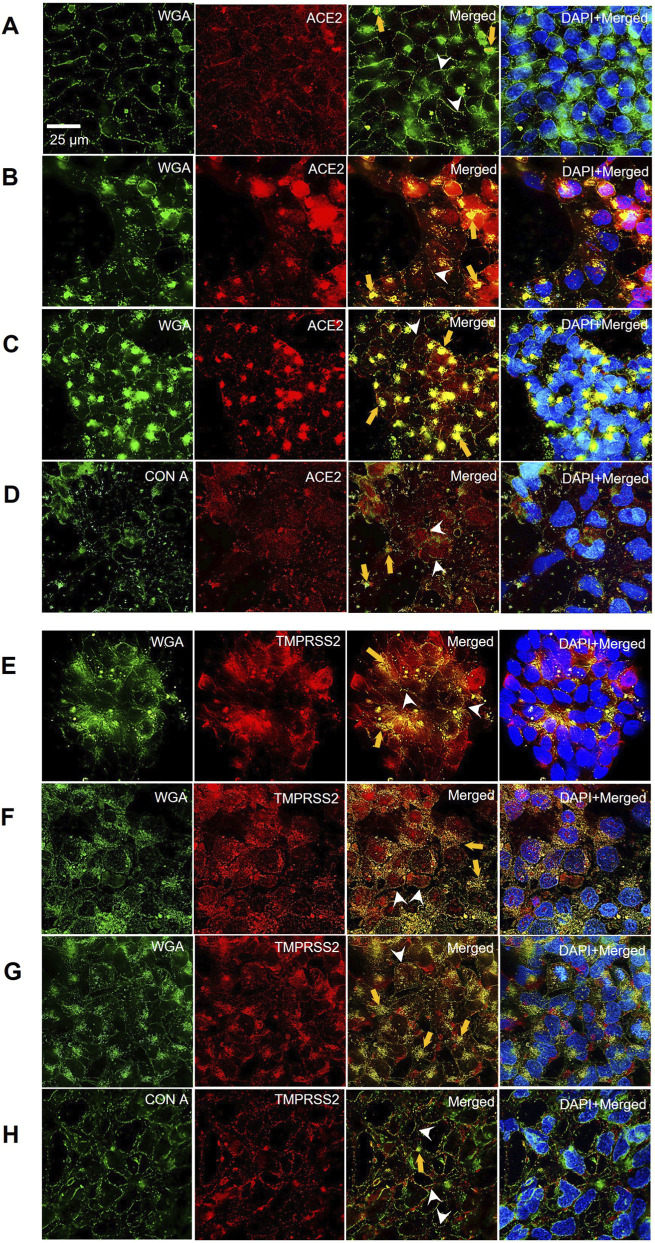
hESCs and their differentiated germ layer cells express the ACE2 receptor and the TMPRSS2 protease. Immunocytochemistry showing ACE2 localization in: **(A)** hESCs, **(B)** endoderm, **(C)** mesoderm, and **(D)** ectoderm. All samples were labeled with fluorescent lectins (WGA-FITC or ConA-FITC) to verify surface localization of ACE2 and TMPRSS2. Merged images of lectin and ACE2 show colocalization. Immunocytochemistry showing TMPRSS2 localization in: **(E)** hESCs, **(F)** endoderm, **(G)** mesoderm, and **(H)** ectoderm. Merged images of lectin and TMPRSS2 show colocalization. White arrowheads show individual protein puncta. Orange arrows show protein clusters. Representative images of three independent experiments are shown.

We first determined if and where the viral entry proteins were located on the hESCs and germ layer cells. Host cells were labeled with lectins that bind cell surface glycans (Figure 1). WGA was localized on the surface of hESCs, endoderm, and mesoderm, and Con A was localized on the surface of ectoderm. The 4 cell types were then labeled with the ACE2 or TMPRSS2 antibodies (Figures 1A–D). The ACE2 and lectin (WGA or Con A) images were merged, and in all 4 cell types, ACE2 antibody was colocalized with the lectin (Figures 1A–D), indicating ACE-2 was present in the plasma membrane. However, differences in distribution of ACE2 were noted among the cell types. In hESCs, ACE2 was mainly distributed as individual proteins (Figure 1A white arrowheads), while some ACE2 was present in small aggregates (Figure 1A orange arrows). In the endoderm and mesoderm, individual ACE2 proteins were present (Figure 1B, C white arrowheads); however, much of the ACE-2 and lectin were localized in numerous large aggregates (Figure 1B, C orange arrows). In the ectoderm, there were individual ACE2 proteins in the plasma membrane (Figure 1D white arrowheads); however, unlike the other germ layers, there was very little aggregation of ACE2 into clusters (Figure 1D orange arrows).

Like ACE2, TMPRSS2 antibody bound to all 4 cell types and was colocalized with lectin, indicating it was present on the cell surface (Figures 1E–H). In hESCs, individual TMPRSS2 proteins (Figure 1E white arrowheads) and small aggregates were observed (Figure 1E orange arrows). In the endoderm and mesoderm, there were both individual TMPRSS2 proteins in the plasma membrane (Figure 1F, G white arrowheads), and numerous large aggregates of TMPRSS2 and lectin (Figure 1F, G orange arrows). In the ectoderm, there were individual TMPRSS2 proteins (Figure 1H white arrowheads), but aggregates were small and sparse (Figure 1H orange arrows).

These data showed that the 4 cell types had the machinery needed for SARS-CoV-2 entry via the fusion or endocytosis pathways. Both ACE2 and TMPRSS2 were less abundant and less clustered in the ectoderm than in the other 3 cell types.

### 3.2 SARS-CoV-2 pseudoparticles preferentially infect ectoderm

**FIGURE 2:**
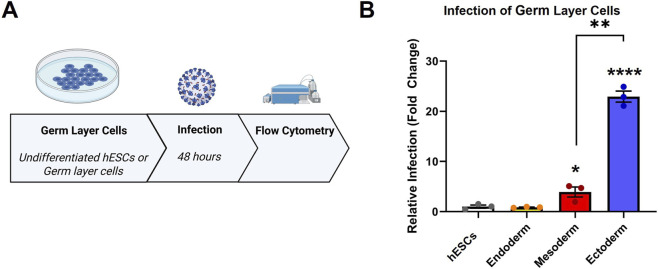
H9 hESCs and their differentiated germ layers were susceptible to SARS-CoV-2 pseudoparticle infection. **(A)** Flow chart showing the overall infection procedure. **(B)** Flow cytometry was performed to check the relative infection in the hESCs and germ layer cells. A one-way ANOVA was performed on raw data divided by the number of cells to account for various seeding densities, and Dunnett’s *post hoc* test was used to compare differentiated groups to the hESCs. Data are the means ± SEM of three independent experiments. * = p < 0.05, ** = p < 0.01.

SARS-CoV-2 pseudoparticles were produced using previously published procedures (Song et al., 2023a; Figure 2A). To test the susceptibility of hESCs and the germ layer cells to SARS-CoV-2 infection, a MOI of 0.1 was used (Supplementary Figure S4). The mean infection in undifferentiated hESCs was set to 1, and the germ layer groups were compared to this value (Figure 2B). Relative infection varied significantly among the 4 cell types. Infection levels were similar for the undifferentiated hESCs and endoderm. However, infection was significantly elevated in both mesoderm and ectoderm. The infection in ectoderm was 23-fold greater than in hESCs and 6-fold greater than in mesoderm. These results show that hESCs and germ layer cells were infected by SARS-CoV-2 pseudoparticles and that ectoderm had a much higher susceptibility than the other 3 cell types.

### 3.3 SARS-CoV-2 pseudoparticles entered ectoderm via the TMPRSS2 membrane fusion pathway

**FIGURE 3:**
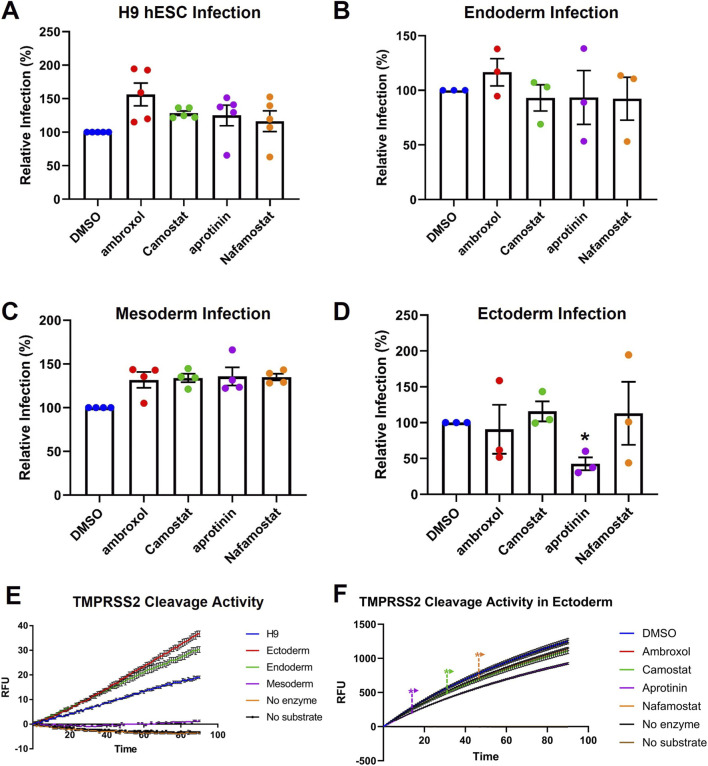
TMPRSS2 inhibitors decreased infection in ectoderm but not in the other cell types. Data are normalized to the DMSO control. SARS-CoV-2 pseudoparticle infection was not significantly affected by TMPRSS2 inhibitors in: **(A)** hESCs, **(B)** endoderm, and **(C)** mesoderm. **(D)** Aprotinin significantly decreased infection in ectoderm. In **(A–D)** one-way ANOVAs were performed on raw data, and Dunnett’s *post hoc* test was used to compare treated groups to the DMSO control. **(E)** TMPRSS2 activity of the hESCs and their germ layers. **(F)** Aprotinin decreased TMPRSS2 cleavage in ectoderm. In **(E)** and **(F)**, RFU = relative fluorescence units. In **(E)**, **(F)**, two-way ANOVAs were performed on raw data. In **(F)**, aprotinin significantly inhibited TMPRSS2 activity starting as early as 13 min and maintained strong inhibition throughout the assay. Camostat and Nafamostat showed significant reduction starting from 33, 45 min, respectively, but the magnitude of inhibition remained marginal compared to that of aprotinin. Ambroxol was not significantly different from DMSO. In **(A–F)**, each group is the mean ± SEM of three independent experiments. * = p < 0.05.

TMPRSS2 inhibitors (ambroxol, Camostat, aprotinin, Nafamostat) were tested to determine if they could reduce viral infection *via* the fusion pathway in hESCs and the germ layer cells. The mean infection in DMSO was set to 100, and the inhibitor groups were compared to this value. Statistical analysis showed that none of the inhibitors were significantly different than the DMSO control in hESCs (Figure 3A), endoderm (Figure 3B), and mesoderm (Figure 3C). However, in ectoderm, aprotinin significantly reduced infection relative to the DMSO control (Figure 3D).

To compare TMPRSS2 activity across cell types, Boc-Gln-Ala-Arg-AMC HCl, a fluorogenic substrate, was added to lysates of hESCs and germ layer cells. Lysates from hESCs, ectoderm, and endoderm cleaved the TMPRSS2 substrate when compared to the negative controls (no enzyme, no substrate) (Figure 3E). While mesoderm had almost no TMPRSS2 activity, ectoderm had the highest activity, which is likely a factor in its higher susceptibility to infection.

Susceptibility to infection could also be due to variations in the potencies of the four inhibitors. Therefore, the ability of each inhibitor to reduce TMPRSS2 activity in ectodermal lysates was tested using the fluorogenic substrate (Boc-Gln-Ala-Arg-AMC HCl) (Figure 3F). Aprotinin significantly reduced protease activity in ectodermal lysates (Figure 3F), consistent with its ability to block infection. In contrast, Ambroxol showed no effect (Ambroxol red line underlies DMSO control). Camostat and Nafamostat significantly inhibited TMPRSS2 activity; however, their inhibition was not as great as that of aprotinin, which may explain their failure to inhibit viral entry in ectoderm.

### 3.4 SARS-CoV-2 pseudoparticles entered hESCs and the germ layer cells via endocytosis

**FIGURE 4:**
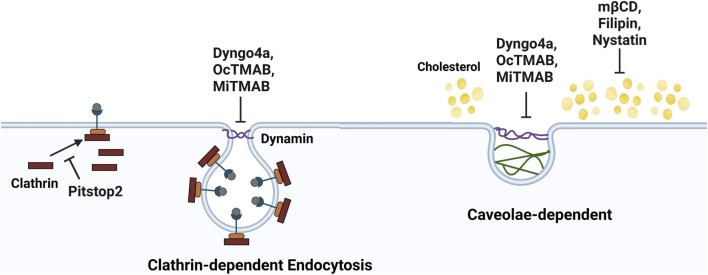
Diagram summarizing the effects of endocytosis inhibitors on SARS-CoV-2 entry. Dynamin-dependent endocytosis includes both clathrin- and caveolae-mediated endocytosis. The clathrin-mediated endocytosis pathway can be blocked by targeting various intermediates. Pitstop2 inhibits clathrin recruitment to the receptor. OcTMAB and MiTMAB blocks dynamin GTPase recruitment and dynamin activity is reduced by Dyngo4a. Caveolae-dependent endocytosis can be blocked by targeting cholesterol. mβCD and nystatin depletes cholesterol from the plasma membrane. Filipin loosens the packing of acyl chains by interacting with cholesterol.

**FIGURE 5:**
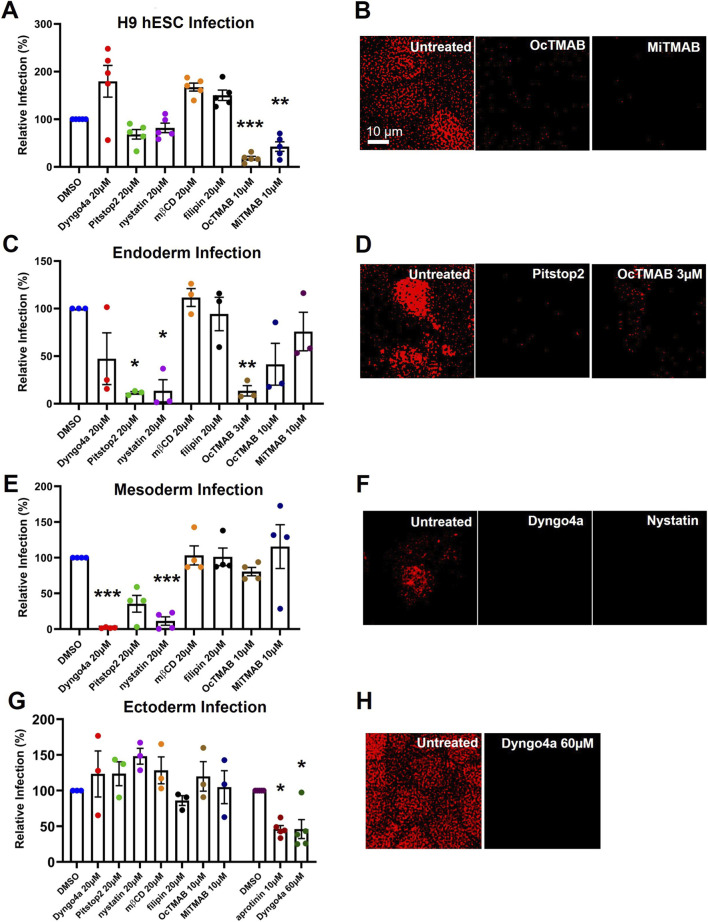
Endocytosis inhibitors decreased infection in all 4 cell types. **(A)** OcTMAB and MiTMAB significantly decreased infection in hESCs. **(B)** OcTMAB and MiTMAB decreased endocytosis of TRITC-conjugated dextran. **(C)** Pitstop2, nystatin, and OcTMAB (3 µM) significantly decreased infection in endoderm. **(D)** Pitstop2 and OcTMAB (3 µM) decreased endocytosis of TRITC-conjugated dextran in endoderm. **(E)** Dyngo4a and nystatin significantly decreased infection in mesoderm. **(F)** Dyngo4a and nystatin decreased endocytosis of TRITC-conjugated dextran in mesoderm. **(G)** Dyngo4a (60 µM) significantly decreased infection in ectoderm. **(H)** Dyngo4a (60 µM) decreased endocytosis of TRITC-conjugated dextran. In **(A)**, **(C)**, **(E)**, **(G)**, one-way ANOVAs were performed on the raw infection data, and Dunnett’s *post hoc* test was used to compare treated groups to the DMSO control. Data are the means ± SEM of three independent experiments. * = p < 0.05, ** = p < 0.01, *** = p < 0.001. In **(B)**, **(D)**, **(F)**, **(H)**, representative images of three independent experiments are shown.

SARS-CoV-2 can also enter host cells by endocytosis (Bayati et al., 2021; Figure 4). Pitstop2, Dyngo4a, OcTMAB, MiTMAB block the clathrin entry pathway, while Nystatin, Dyngo4a, OcTMAB, MiTMAB block the caveolae pathway (as shown in Figure 4). The mode of SARS-CoV-2 entry in germ layer cells was also tested with these endocytosis inhibitors to determine if they could reduce infection in hESCs and the germ layer cells relative to the DMSO control. In hESCs, Pitstop2, nystatin, OcTMAB, and MiTMAB reduced infection relative to DMSO, but only OcTMAB and MiTMAB were significant (Figure 5A). In the untreated control, dextran-TRITC was taken up by the hESCs and appeared as fluorescent puncta, consistent with uptake by endocytosis (Figure 5B). However, hESCs treated with OcTMAB or MiTMAB showed little or no uptake of dextran-TRITC.

Pitstop2 (20 µM), nystatin (20 µM), and OcTMAB (3 µM) significantly decreased infection in endodermal cells (Figure 5C). Dyngo4a (20 µM) and OcTMAB (10 µM) also reduced infection but were not significantly different from the DMSO control. Nystatin treatment caused cell death, so only Pitstop2 and OcTMAB (3 µM) were tested for dextran-TRITC uptake (Figure 5D). Endodermal cells treated with Pitstop2 or OcTMAB showed little or no fluorescence compared to the untreated control.

In mesoderm, Dyngo4a (20 µM), Pitstop2 (20 µM), nystatin (20 µM), and OcTMAB (10 µM) decreased infection relative to the DMSO control, but only Dyngo4a and nystatin were significant (Figure 5E). Both Dyngo4a and nystatin prevented the uptake of dextran-TRITC compared to the untreated control (Figure 5F).

The endocytosis inhibitor concentrations used above did not block viral pseudoparticle infection of ectoderm. In a subsequent screen using higher concentrations of the inhibitors, only Dyngo4a reduced infection in ectoderm (Figure 5G). Therefore, an additional experiment was run using Dyngo4a at 60 μM, which significantly reduced infection of ectodermal cells (Figure 5G). Dextran-TRITC uptake was completely prevented by Dyngo4a (60 µM) compared to the untreated control (Figure 5H).

### 3.5 Endocytosis inhibitors did not alter most germ layer markers

To determine if the drugs that inhibited infection affected germ layer differentiation, qPCR was performed using markers for each cell type. In undifferentiated hESCs, *OCT4* expression was not significantly different in the untreated control and inhibitor treated groups (Supplementary Figure S5A). In the endoderm, Pitstop2 did not significantly affect *SOX17* expression; however, significant upregulation was observed with OcTMAB (Supplementary Figure S5B). In the mesoderm, Dyngo4a did not significantly affect *NCAM* expression, but *NCAM* was significantly upregulated with nystatin (Supplementary Figure S5C). In the ectoderm, the expression of *PAX6* was not significantly different from the control in the aprotinin and Dyngo4a treated groups (Supplementary Figure S5D).

### 3.6 Cell surface glycosylation affected tropism

The observed variation in SARS-CoV-2 pseudoparticle infectability among the 4 cell types may have be partially attributed to elevated TMPRSS2 activity in the ectoderm and the use of both the membrane fusion and endocytosis entry pathways by ectodermal cells. In contrast, the other cell types—hESCs, endoderm, and mesoderm—seemed to rely exclusively on endocytosis. To further investigate the basis of this tropism, we hypothesized that ectodermal cells exhibit reduced surface glycosylation compared to the other 3 cell types, thereby facilitating greater accessibility of the spike protein to the ACE2 receptor on the plasma membrane.

**FIGURE 6:**
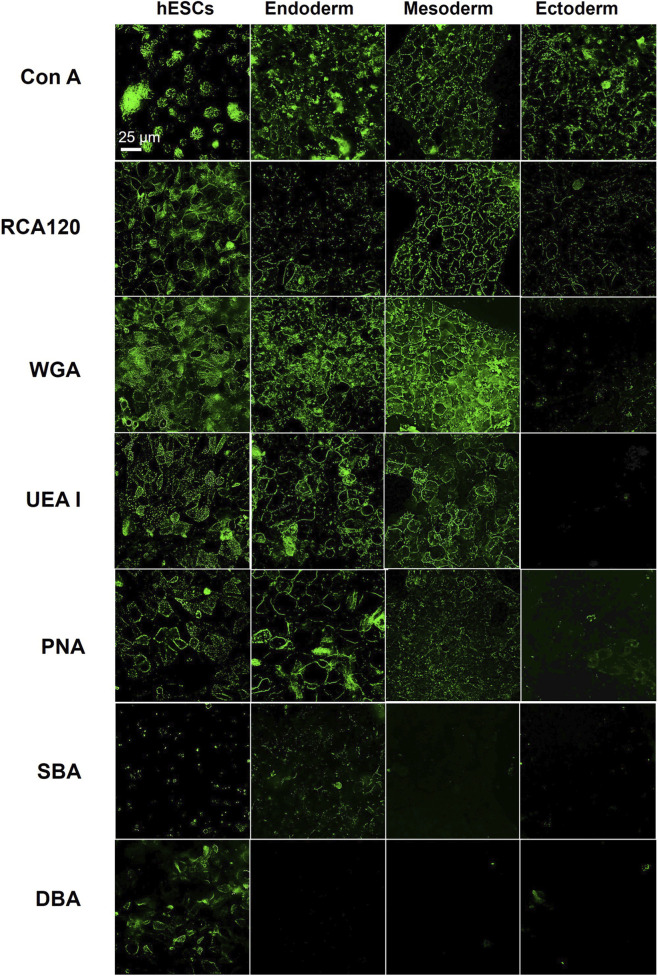
hESCs and the three germ layers showed differential glycosylation when labeled with FITC-conjugated lectins. Various FITC-lectins (ConA, RCA120, WGA, UEA I, PNA, SBA, DBA) were used to label hESCs and the germ layer cells. Of the seven lectins, only ConA bound to the ectoderm, while the other 3 cell types were well labeled by five (ConA, RCA120, WGA, UEA1 and PNA) of the seven lectins.

To test this hypothesis, the 4 cell types were labeled with seven FITC-conjugated lectins that bind a range of cell surface sugar moieties (Figure 6). Glycosylation patterns varied across cell types. Con A bound well to all cell types. RCA120 bound to all cell types with weaker signal in the endoderm and ectoderm. SBA bound weakly to hESCs and endoderm. DBA bound to hESCs only. The remaining lectins (WGA, UEA I, PNA) bound to hESCs, endoderm, and mesoderm but showed no detectable binding to the ectoderm. These results show differential glycosylation among the 4 cell types and support the hypothesis that ectodermal cells are comparatively less glycosylated.

**FIGURE 7:**
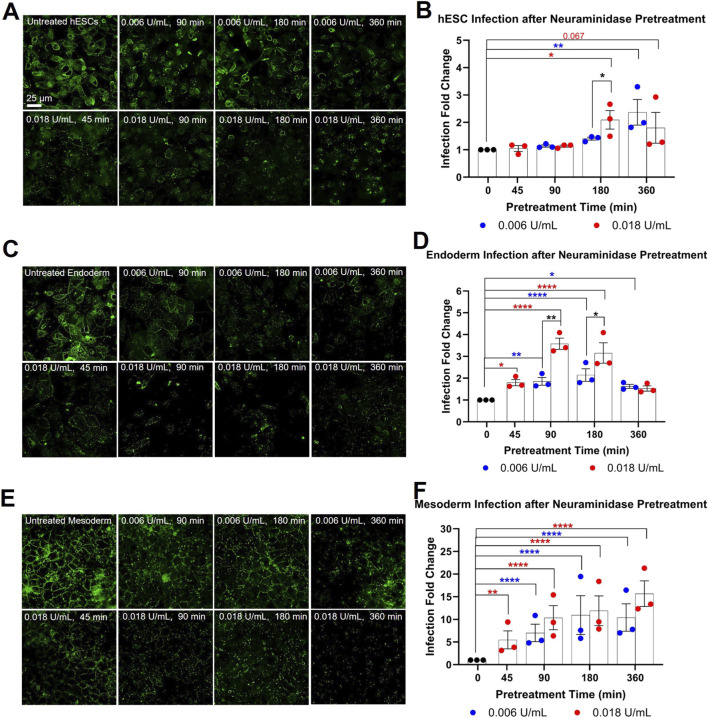
Neuraminidase treatment of hESCs, endoderm, and mesoderm increased infection. **(A)** H9 hESCs, **(C)** endoderm, and **(D)** mesoderm were labeled using FITC-WGA with or without neuraminidase treatment (0.006 U/mL, 0.018 U/mL). Treated cells showed loss of WGA-FITC labeling *versus* the untreated control. Neuraminidase treatment significantly increased infection in: **(B)** hESCs, **(D)** endoderm, and **(F)** mesoderm, based on one-way ANOVAs on raw infection data followed by Dunnett’s *post hoc* tests to compare neuraminidase treated groups to the untreated control. An unpaired two-tailed t-test was used to compare 0.006 U/mL and 0.018 U/mL groups against each other. Data are the means ± SEM of three independent experiments. * = p < 0.05, ** = p < 0.01, **** = p < 0.0001. Blue asterisk = one-way ANOVA significance for 0.006 U/mL of neuraminidase; Red asterisk = one-way ANOVA significance for 0.018 U/mL of neuraminidase; Black asterisk = t-test significance for 0.006 U/mL and 0.018 U/mL comparisons.

To further test this hypothesis, sialic acid, which binds WGA, was enzymatically removed from cell surfaces of hESCs, endoderm, and mesoderm with neuraminidase, and SARS-CoV-2 pseudoparticle infection was compared to untreated controls. Sialic acid was chosen for removal because WGA binding was elevated in hESCs, endoderm, and mesoderm, but not observed in ectoderm (Figure 6). Cells were incubated with 0.006 U/mL or 0.018 U/mL of neuraminidase for 0, 45, 90, 180, or 360 min to remove surface sialic acid. Neuraminidase treatment reduced WGA-FITC binding to hESCs, endoderm, and mesoderm (Figures 7A,C,E), indicating the removal of most sialic acid groups from the cell surfaces. WGA binding decreased as incubation time increased (e.g., compare 0.006 U/mL in hESCs at 90, 180, and 360 min) and as enzymatic activity increased (e.g., compare hESCs treated with 0.006 vs. 0.018 U/mL at 90 min) (Figure 7A).

To determine if removal of sialic acid would increase infection of hESCs, cells were pretreated with neuraminidase (0.006 U/mL, 0.018 U/mL) for various periods of time before infecting them with SARS-CoV-2 pseudoparticles. In hESCs, a significant increase in infection was observed in the group pretreated for 180 min with 0.018 U/mL of neuraminidase, and a similar increase in infection occurred in the group pretreated for 360 min with 0.006 U/mL of neuraminidase (Figure 7B). In the groups pretreated for 180 min with neuraminidase, 0.018 U/mL produced greater infection than 0.006 U/mL. The maximum increase in infection for the hESCs treated with neuraminidase was slightly over 2-fold, which was not as high as the 23-fold increase observed in the ectoderm (Figure 2B). Together, the decrease in WGA labeling correlated with an increase in infection in hESCs.

Endoderm was very susceptible to neuraminidase treatment. WGA labeling was decreased at all times and by both concentrations of neuraminidase (Figure 7C), and infection was increased following pretreatment with both concentrations of neuraminidase (Figure 7D). Pretreatment with 0.006 U/mL of neuraminidase increased infection significantly in all groups. Pretreatment with 0.018 U/mL of neuraminidase significantly increased infection in the 45, 90, 180 min groups; however, in the 360 min group, infection was not significantly different than the control, probably because some cell death occurred. In groups pretreated with neuraminidase for 90 min and 180 min, 0.018 U/mL produced significantly greater infection than 0.006 U/mL. Infection of deglycosylated mesoderm was 2–3.5 times greater than in untreated controls.

For mesoderm, neuraminidase treatment decreased WGA-FITC labeling in all treated groups (Figure 7E). Pretreatment with 0.006 U/mL of neuraminidase decreased WGA binding as time of pretreatment increased, while pretreatment with 0.018 U/mL of neuraminidase noticeably decreased WGA binding even at shorter pretreatment time. Infection of mesoderm was significantly increased as time of pretreatment with neuraminidase increased, with the higher concentration producing a larger effect faster (Figure 7F). Pretreatment with 0.006 U/mL gave a 10-fold maximum increase in infection in both the 180 min and 360 min groups. Pretreatment with 0.018 U/mL of neuraminidase gave a 16-fold maximum increase in infection, which was close to the ectoderm infection levels (∼23-fold) (Figure 2B). Overall, in both the endoderm and mesoderm, a positive correlation between the loss of WGA staining and an increase in infection was observed, with the mesoderm being especially responsive to removal of sialic acid.

## 4 Discussion

**FIGURE 8:**
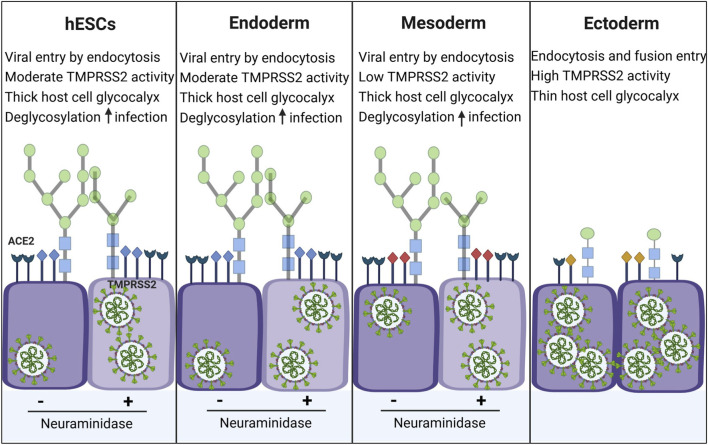
Entry pathways and reasons for tropism in hESCs and germ layer cells. Greater infection of the ectoderm was likely due to three factors: (1) hESCs, endoderm, and mesoderm used only the endocytosis-based pathway for entry into the host cell, while ectoderm used both pathways; (2) TMPRSS2 activity was highest in ectoderm, which likely facilitated its infection by the membrane fusion pathway; (3) hESCs, endoderm and mesoderm had heavier glycosylation than ectoderm, and removal of sialic acid with neuraminidase, increased the infectability of hESCs, endoderm, and mesoderm.

*In vitro* models are well suited for identification of viruses with embryo-lethal or teratogenic potential, especially when *in vivo* studies are ethically or practically unfeasible (Talbot, 2008; Talbot and Lin, 2011a; b; Lin et al., 2021). In this study, we used a “disease-in-a-dish” model to characterize the susceptibility of hESCs and the three germ layers to infection by SARS-CoV-2 pseudoparticles (Song et al., 2023a; b). Our data demonstrate that cells representative of early human embryonic development are susceptible to SARS-CoV-2 infection, *in vitro*. These findings suggest that if vertical transmission occurs during this critical window, the virus could directly target embryonic tissues. Of the cell types tested, ectodermal cells had the highest susceptibility to infection, which is likely multifactorial and may be attributed to: (1) the use of both membrane fusion and endocytosis entry pathways, in contrast to apparent reliance on endocytosis by hESCs, endoderm, and mesoderm; (2) elevated TMPRSS2 activity in ectodermal cells, which could enhance S2 cleavage and membrane fusion; and (3) reduced surface glycosylation on ectoderm, which could facilitate access of viral spike protein to its ACE2 receptor on the host cell plasma membrane (Figure 8). These mechanistic insights provide a plausible explanation for the observed tissue tropism and suggest ectodermal derivatives, specifically the nervous system and epidermis, may be vulnerable to SARS-CoV-2-mediated injury.

SARS-CoV-2 entry by membrane fusion involves spike protein, ACE2, and TMPRSS2 (Cai et al., 2020; Hoffmann et al., 2020). We tested four TMPRSS2 inhibitors that were previously studied *in vitro* with SARS-CoV-2 or administered to COVID-19 patients (Wang et al., 2023; Carpinteiro et al., 2021; Olaleye et al., 2020; Hoffmann et al., 2020; Hoffmann et al., 2021; Li et al., 2021; Lu et al., 2022; Song et al., 2023b). At a concentration of 10 μM, only aprotinin significantly reduced SARS-CoV-2 pseudoparticle infection, and its effect was restricted to ectodermal cells, where it inhibited both viral entry and TMPRSS2 activity. Our results align with previous studies showing that aprotinin at a similar concentration (20 µM) inhibited SARS-CoV-2 infection in Calu-3 and human primary epithelial cells (Bojková et al., 2020; Bestle et al., 2020). Moreover, in a clinical trial, inhaled aprotinin significantly reduced viral load in patients with mild to moderate COVID-19 symptoms (Redondo-Calvo et al., 2022).

In contrast, results with other TMPRSS2 inhibitors are mixed. Infection of Vero E6 cells was inhibited by 0.1–10 µM concentrations of ambroxol (Olaleye et al., 2020). However, ambroxol (10 µM) did not inhibit infection of HEK293T and A549 (Wang et al., 2023). In agreement with Wang et al. (2023), in our study, ambroxol did not inhibit infection of human embryonic cells, and this was due to its failure to significantly inhibit TMPRSS2 activity. At higher concentrations (100–1000 µM), significant inhibition was observed in HEK293T and A549 cells (Wang et al., 2023), which may reflect non-specific mechanisms, such as acid sphingomyelinase inhibition (Carpinteiro et al., 2021) or enhanced mucus secretion (Zanasi et al., 2017). Clinically, ambroxol did not decrease mortality in hospitalized COVID-19 patients (Lu et al., 2022), while bromhexine, its parent drug, significantly reduced the rate of ICU admissions, intubation/mechanical ventilation, and mortality in patients with COVID-19 (Ansarin et al., 2020).

Camostat and nafamostat also yielded variable results. Both small molecules inhibited SARS-CoV-2 infection in Calu-3 lung cells at 20 µM (Hoffmann et al., 2020; 2021) and in human airway epithelial cells at 25 µM (Li et al., 2021). However, these concentrations may elicit off-target effects, including inhibition of enteropeptidase (Sun et al., 2020), suppression of cytokine release (Hoffmann-Winkler et al., 2020), and anticoagulant activity (Qian et al., 2023). In our study, embryonic cells were unresponsive to 10 µM camostat or nafamostat, although 5 µM was effective in human lung tissue (Hoffmann et al., 2021), supporting the idea that protease inhibitor efficacy is tissue-specific (Song et al., 2023b). Case reports suggest that nafamostat may alleviate early symptoms (Doi et al., 2020; Takahashi et al., 2021), but its lack of efficacy in hospitalized patients (Hoffmann-Winkler et al., 2020; Gunst et al., 2021) parallels findings with ambroxol, suggesting that therapeutic responsiveness is stage-dependent.

Taken together, the efficacy of these protease inhibitors varied with cell type and concentration in both *in vitro* and clinical studies. Notably, aprotinin emerged as the most promising candidate for prenatal applications, not only because of its efficacy in blocking TMPRSS2-mediated fusion in ectodermal cells but also because of its favorable safety profile. Aprotinin is classified as an FDA Pregnancy Category B drug, indicating that animal reproduction studies have failed to demonstrate a risk to the fetus. Unlike other inhibitors, such as ambroxol or camostat, which have mixed safety or efficacy records, aprotinin has shown no evidence of teratogenicity or embryotoxicity in animal models. This safety profile, combined with our efficacy data, supports its prioritization for further investigation in the context of COVID-19 in pregnancy.

SARS-CoV-2 can also enter host cells by clathrin and caveolae-mediated endocytosis (Bayati et al., 2021). In some cells, such as mink lung epithelium which has an inactive form of TMPRSS2, endocytosis is the only entry pathway (Song et al., 2023b). In the current study, all cell types used the endocytosis entry pathway, but inhibitors that were effective varied across cell types. Pitstop2 (20 µM) inhibited infection in hESCs, endoderm, and mesoderm, in agreement with studies similar to ours using A549 cells, human endothelial cells, and HEK293T cells at concentrations between 10–15 µM (Qu et al., 2024; Qian et al., 2021; Bayati et al., 2021). In our study, low (20 µM) and high (60 µM) concentrations of Pitstop2 had no effect on ectoderm (data not shown), while efficacy at 50 µM in human renal cells (Somova et al., 2024) may be due to nonspecific effects, such as disruption of the nuclear permeability barrier leading to cytotoxicity (Liashkovich et al., 2015).

Although nystatin was predicted to be a good endocytosis inhibitor (Virág et al., 2021) and it (20 µM) inhibited infection of mesodermal cells, it was cytotoxic to endoderm, enhanced infection of VeroE6 cells (Nguyen et al., 2024), and has nonspecific antifungal effects (Baek et al., 2013). These factors combined with a lack of clinical studies suggest that nystatin may not be suitable for use with SARS-CoV-2 research or treatment.

OcTMAB and MiTMAB inhibit clathrin- and caveolae-mediated endocytosis by blocking recruitment of dynamin GTPase to the plasma membrane (Figure 4). In our study, OcTMAB had significant efficacy with hESCs and endoderm, and MiTMAB inhibited entry in hESCs. Concentration is critical *in vivo* as its analog (cetyltrimethylammonium bromide) was embryotoxic and teratogenic in pregnant mice given 10 mL/kg orally (Isomaa and Ekman, 1975). Despite the absence of patient trials, our results indicate that OcTMAB and MiTMAB exhibit efficacy and may be suitable for translation.

Dyngo4a, a dynamin inhibitor, did not affect hESCs, but produced variable efficacy across the germ layers. It was moderately effective in endoderm and ectoderm, and completely blocked pseudoparticle entry in mesoderm, which fits well with the observation that mesoderm did not have TMPRSS2 activity, and therefore used only endocytosis. Our data are corroborated by reports that similar concentrations (12.5–40 µM) of Dyngo4a had efficacy with human endothelial and Caco-2 cells (Qian et al., 2021; Sun et al., 2024). Our effective concentration for the ectoderm (60 µM) was probably not due to nonspecific effects as both infection and dextran-TRITC uptake were blocked and similar concentrations were effective with human renal cells (50 µM) and HEK293T cells (80 µM) (Somova et al., 2024; Bayati et al., 2021), confirming the cell-type-specific nature of inhibitor efficacy.

The main conclusion from our endocytosis inhibitor data is that the efficacy of endocytosis inhibitors varies among cell types, like the variability observed with TMPRSS2 inhibitors (Supplementary Table S2). This cell-type-specific variation in drug sensitivity reflects the underlying differences in the molecular composition and inhibitor potency within the cellular microenvironment, rather than a lack of conservation in the viral entry mechanism itself. While the core infection machinery (ACE2, TMPRSS2) remains conserved, the differential effects of inhibitors can be observed across tissues. For instance, although ectoderm exhibits high TMPRSS2 activity, our data show that Camostat and Nafamostat did not significantly inhibit this protease activity compared to aprotinin. Additionally, the relative contributions of membrane fusion versus endocytosis, the abundance and aggregation state of receptors, and the density of cell surface glycosylation further modulate susceptibility. Thus, effective inhibition requires both the reliance of the target cell on a specific entry pathway and the functional capacity of the drug to act within that specific cellular environment. Although TMPRSS2 inhibitors have been extensively studied, there is a lack of clinical data on endocytosis inhibitors in COVID-19 patients.

Our observations of ACE2 and TMPRSS2 aggregates suggest complex regulation of these viral entry receptors that may impact infection efficiency. We observed a correlation between receptor aggregation and the preferred viral entry pathway: hESCs, endoderm, and mesoderm, which displayed prominent ACE2 and TMPRSS2 aggregates and exclusively utilized endocytosis-mediated entry. This aligns well with recent findings that the aggregation of cell surface proteins can trigger their internalization and lysosomal degradation, a process known as aggregation-dependent endocytosis (Paul et al., 2023). In contrast, ectodermal cells displayed minimal aggregation, with predominantly individual protein puncta, and supported both endocytosis and membrane fusion entry pathways. While further studies are required to determine the functional significance of these aggregates, our data indicate a potential link between receptor aggregation and the restriction of viral entry to the endocytic pathway.

Our data indicate that cell surface glycosylation is a critical determinant in SARS-CoV-2 infectability. hESCs, endoderm, and mesoderm exhibited heavy binding to ConA, RCA120, WGA, and UEA I, whereas ectoderm bound exclusively to ConA. Enzymatic removal of sialic acid from hESCs, endoderm, and mesoderm significantly increased their susceptibility to SARS-CoV-2 pseudoparticles, supporting the conclusion that the complexity and thickness of the glycocalyx play a protective role in host cell infectability by modulating viral access. Because the sugar architecture on each cell type is complex and different (Furukawa et al., 2017), the effects of neuraminidase varied among cell types. While studies have documented the role of viral glycosylation in receptor recognition, research on host cell glycosylation and its impact on viral entry remains limited. Our glycosylation data agree and extend the computational modeling study, which concluded that the glycocalyx imposes a steric hindrance that the SARS-CoV-2 must overcome to engage the ACE2 receptor (Acosta-Gutierrez et al., 2022). Taken together, our data emphasize the importance of glycosylation of host cells in SARS-CoV-2 infectability and explain the high infectability observed in ectodermal cells.

The high susceptibility of ectodermal cells to SARS-CoV-2 infection raises concern that the embryonic and fetal nervous system may be vulnerable in pregnant women with COVID-19. Because ectodermal infection occurs early in development, viral exposure may result in early embryonic loss, potentially before pregnancy is clinically recognized. If the embryo survives but sustains damage, SARS-CoV-2 could act as a teratogen, disrupting the development of ectodermal derivatives. Several viruses are established teratogens: Zika virus, for example, causes microcephaly and under development of the cerebral cortex and the cerebellum (Brasil et al., 2016; Phan and Holland, 2021), while CMV is a leading cause of birth defects in the U.S. (Thackeray et al., 2014), associated with low birth weight, seizures, rashes, and microcephaly (Adams Waldorf and McAdams, 2013; Thackeray et al., 2014), as well as progressive hearing loss during childhood (Manicklal et al., 2013; Lim and Lyall, 2017).

Evidence linking maternal SARS-CoV-2 infection to congenital anomalies remains limited. While a recent systematic review reported increased risks (e.g., preterm birth and low birth weight) in newborns of COVID-19 infected women (El-Atawi et al., 2024), some claim that vertical transmission is unlikely to occur (El-Atawi et al., 2024; Chaubey et al., 2021). In a cohort study of pregnancies exposed to SARS-CoV-2 from 6 weeks preconception to 19 weeks gestation, Calvert et al. (2023) found no increase in major nervous system anomalies (Odds Ratio = 1.02), including neural tube defects, hydrocephaly, microcephaly, arhinencephaly, holoprosencephaly, and agenesis of the corpus callosum. While these gross anatomical deficits are easily detectable, embryo loss early in pregnancy or subtle anomalies later in development would have been missed. Like CMV, congenital defects from SARS-CoV-2 infection could be latent at birth and appear during childhood. Longitudinal monitoring of children born to mothers with COVID-19 is essential to determine whether delayed neurological deficits or other developmental abnormalities emerge over time.

In adults, COVID-19 symptoms are not limited to the respiratory system, frequently affecting both the central and peripheral nervous systems, derivatives of the ectoderm (Guerrero et al., 2021). Neurological manifestation during SARS-CoV-2 infection include a loss of taste and smell (Saniasiaya et al., 2021; Agyeman et al., 2020; Spudich and Nath, 2022), Guillain-Barre syndrome (an autoimmune condition of the peripheral nerves) (Toscano et al., 2020; Rahimi, 2020), and encephalopathy (a broad term for damage or disease that affects the brain) (Poyiadji et al., 2020; Siahaan et al., 2022). In “long COVID”, patients commonly report fatigue, “brain fog” (e.g., confusion, poor memory, and poor concentration), shortness of breath, and sleep disorder (Perlis et al., 2022; Alkodaymi et al., 2022). Our findings demonstrate SARS-CoV-2 tropism for ectodermal cells during early prenatal development, suggesting that ectodermal derivatives may be intrinsically susceptible to infection. Whether a similar tropism underlies the neurological sequelae observed in adults remains an open question. Future studies should investigate whether the increased susceptibility of ectodermal tissues observed in embryonic models is recapitulated in adult neural and epithelial systems, potentially offering mechanistic insight into the diverse neurological outcomes of COVID-19.

In conclusion, the major findings of our study were that: (1) human embryonic cells (epiblast and germ layer cells) were susceptible to SARS-CoV-2 infection, (2) cell types varied in infectability with ectoderm being the most susceptible; and (3) the increased susceptibility of ectoderm was due to its use of two entry pathways (fusion and endocytosis), higher TMPRSS2 activity, and a much reduced glycocalyx that facilitated interaction of the viral spike protein and ACE2 receptor. These findings suggest that early ectodermal infection may compromise the development of neural and cutaneous tissues, potentially leading to latent or subclinical anomalies not evident at birth. Clinical surveillance of newborns, toddlers, and children exposed to SARS-CoV-2 *in utero* is warranted to identify potential neurodevelopmental deficits.

Our data further support endocytosis as a viable entry route for SARS-CoV-2 in hESCs and germ layer cells. Notably, endocytosis inhibitors, though less studied than TMPRSS2 inhibitors, showed variable efficacy across cell types. The combined use of aprotinin and Dyngo4a may offer a synergistic blockade of both entry pathways. In a clinical setting, the primary objective of inhibitor therapy would be to reduce the maternal viral load, thereby limiting transplacental transmission. However, should the virus breach the placental barrier, our data suggest that these small molecule inhibitors, many of which can cross the placenta (Mandelbrot et al., 2014), could offer a secondary line of defense at the embryonic tissue level. Supporting this possibility, existing pharmacokinetic studies on protease inhibitors, such as darunavir and lopinavir, demonstrate that small molecules can cross the human placenta (Mandelbrot et al., 2014; Pacifici, 2005), suggesting that aprotinin and endocytosis inhibitors could also potentially reach the fetal compartment. These findings highlight the translational potential of repurposed inhibitors in mitigating the risks of vertical transmission and fetal injury.

### 4.1 Limitations of the study

While hESC-derived germ layers faithfully recapitulate the expression of key developmental markers and the entry machinery (ACE2, TMPRSS2) found in embryos *in vivo*, this model lacks the protective placental barrier and maternal immune system. This study tested a single MOI, whereas *in vivo*, the number of viral particles reaching the embryo during pregnancy is likely to vary among patients and may influence infection outcomes. The fluorescent-based peptide substrate used to assess protease activity is not fully specific for its intended target, TMPRSS2 (Lauer-Fields et al., 2001; Zou et al., 2023). Several drugs used to identify the viral entry pathway are FDA approved or are currently in clinical trials (Ambroxol, Camostat, and Dyngo4a). Although these compounds hold promise for preventing infection in pregnant individuals and their embryos/fetuses, none demonstrated universal efficacy across all cell types tested. Furthermore, although our *in vitro* data demonstrate efficacy, the *in vivo* pharmacokinetics and placental transfer rates of these specific inhibitors in the context of COVID-19 remain to be established. Future studies could test mixtures of drugs over a range of doses and look for drug interactions leading to synergy, additivity, and antagonism, while also assessing safety and tissue-specific delivery *in vivo*.

## Data Availability

The original contributions presented in the study are included in the article/Supplementary Material, further inquiries can be directed to the corresponding author.
