# Supplementary material for: SARS-CoV-2 pseudoparticles preferentially infect ectoderm in human embryonic tissues
Source: Front Cell Dev Biol. 2026 Apr 7;14:1733662. doi: 10.3389/fcell.2026.1733662 (PMC13095763; doi:10.3389/fcell.2026.1733662)
Supplement: Supplementary file 1 [file DataSheet1.pdf]

Supplementary Material

1 Supplementary Figures and Tables

1.1 Supplementary Figures

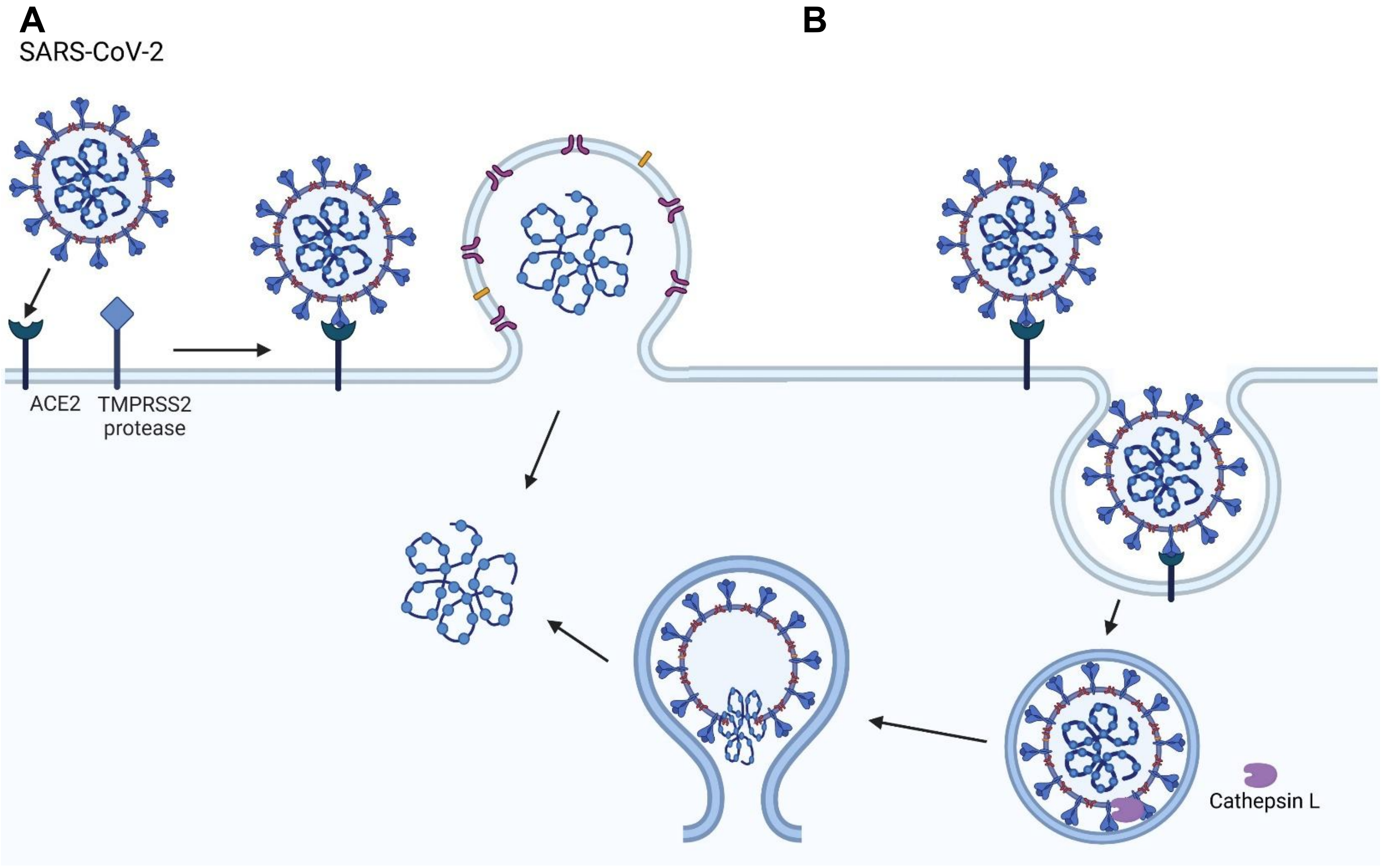

Supplementary Figure 1. SARS-CoV-2 viral entry mechanisms. The SARS-CoV-2 virus binds to the ACE2 host cell receptor then either (A) TMPRSS2 processing of the viral spike protein leads to the fusion of the virus to the cell membrane or (B) the virus is taken up by endocytosis.

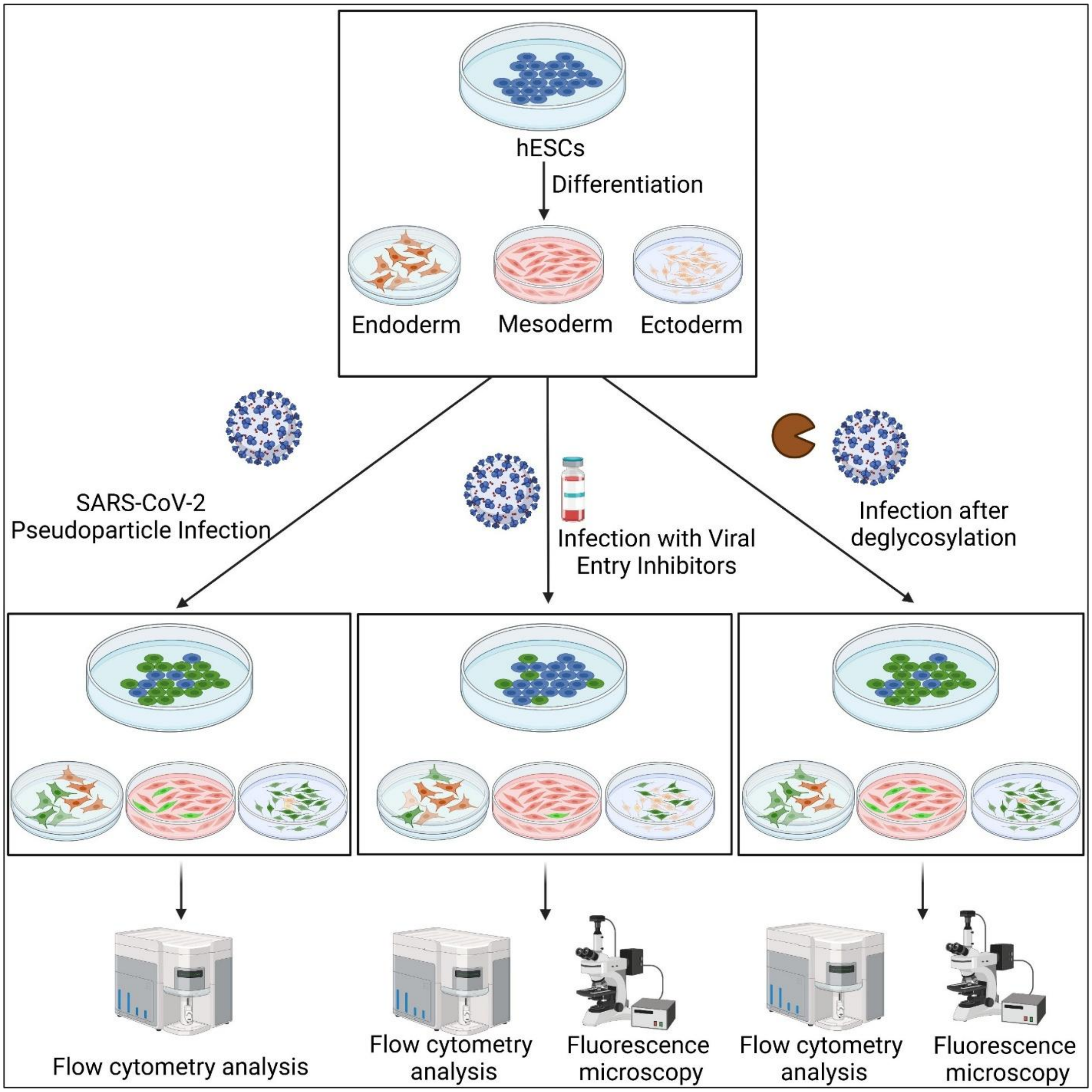

Supplementary Figure 2. A diagram showing the overall experimental set up for the "disease-in-a-dish" model to investigate SARS-CoV-2 infection during early embryonic development.

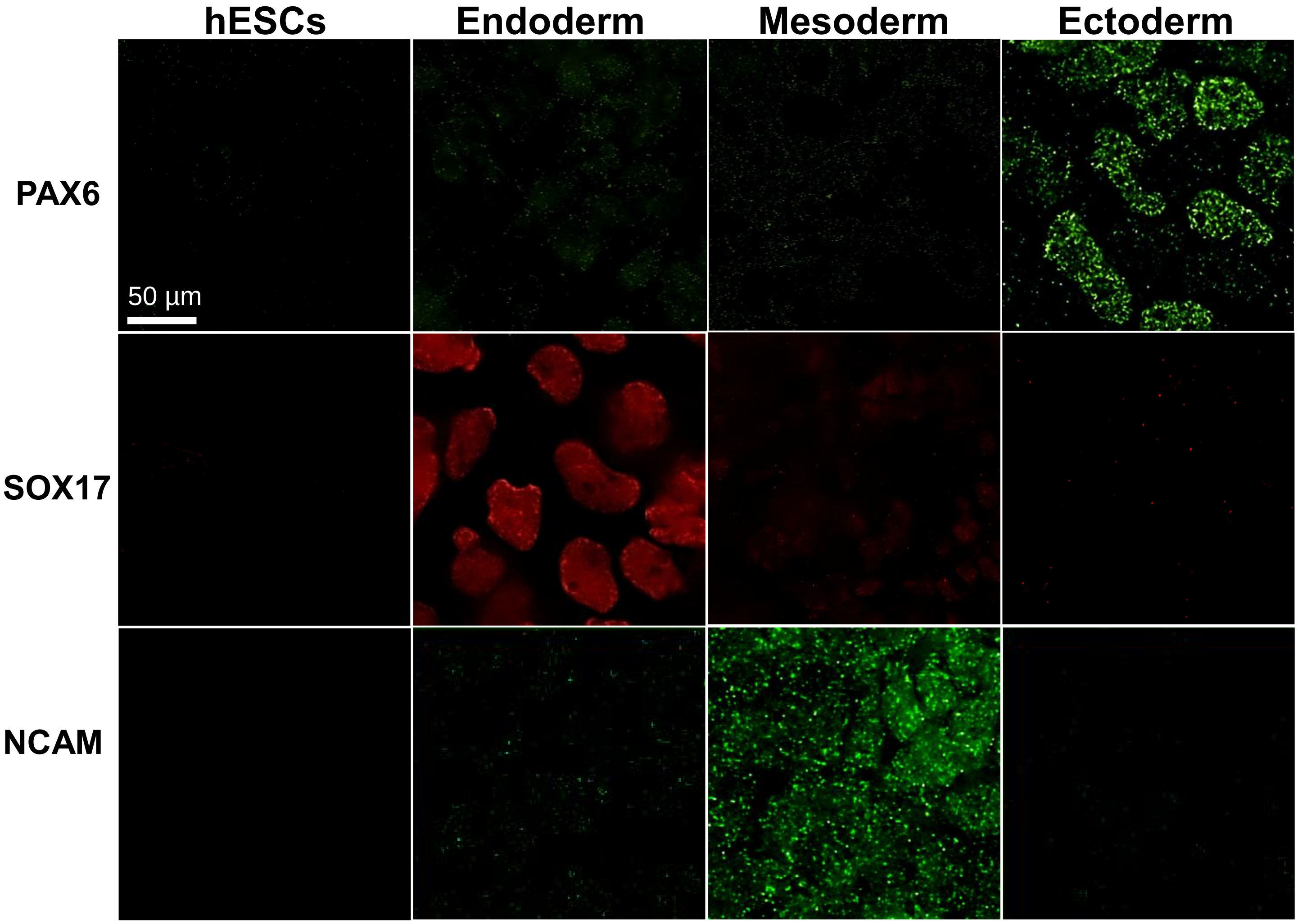

Supplementary Figure 3. The three germ layers were differentiated from H9 hESCs. Immunocytochemistry showing successful differentiation of H9 hESCs into endoderm, mesoderm, and ectoderm.

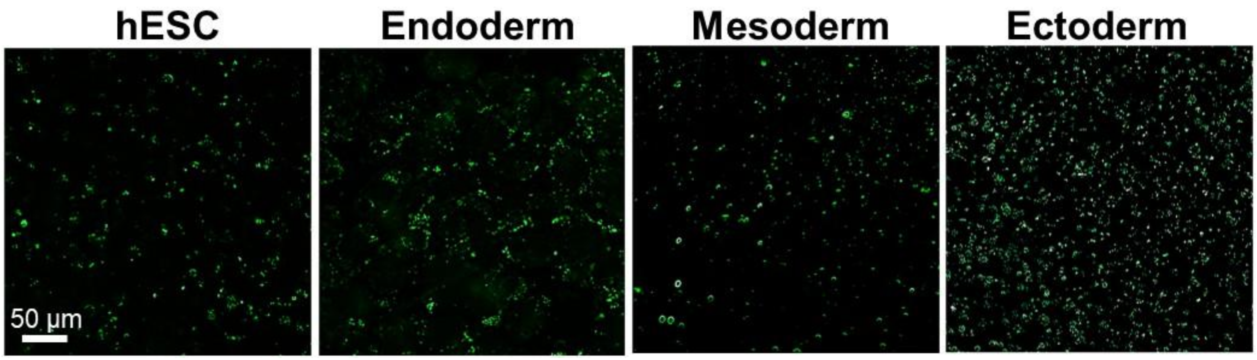

Supplementary Figure 4. Representative fluorescent images of SARS-CoV-2 pseudoparticle infected H9 hESCs and the three germ layers. hESCs and the germ layers were Zsgreen-positive, indicating infection with SARS-CoV-2 pseudoparticles.

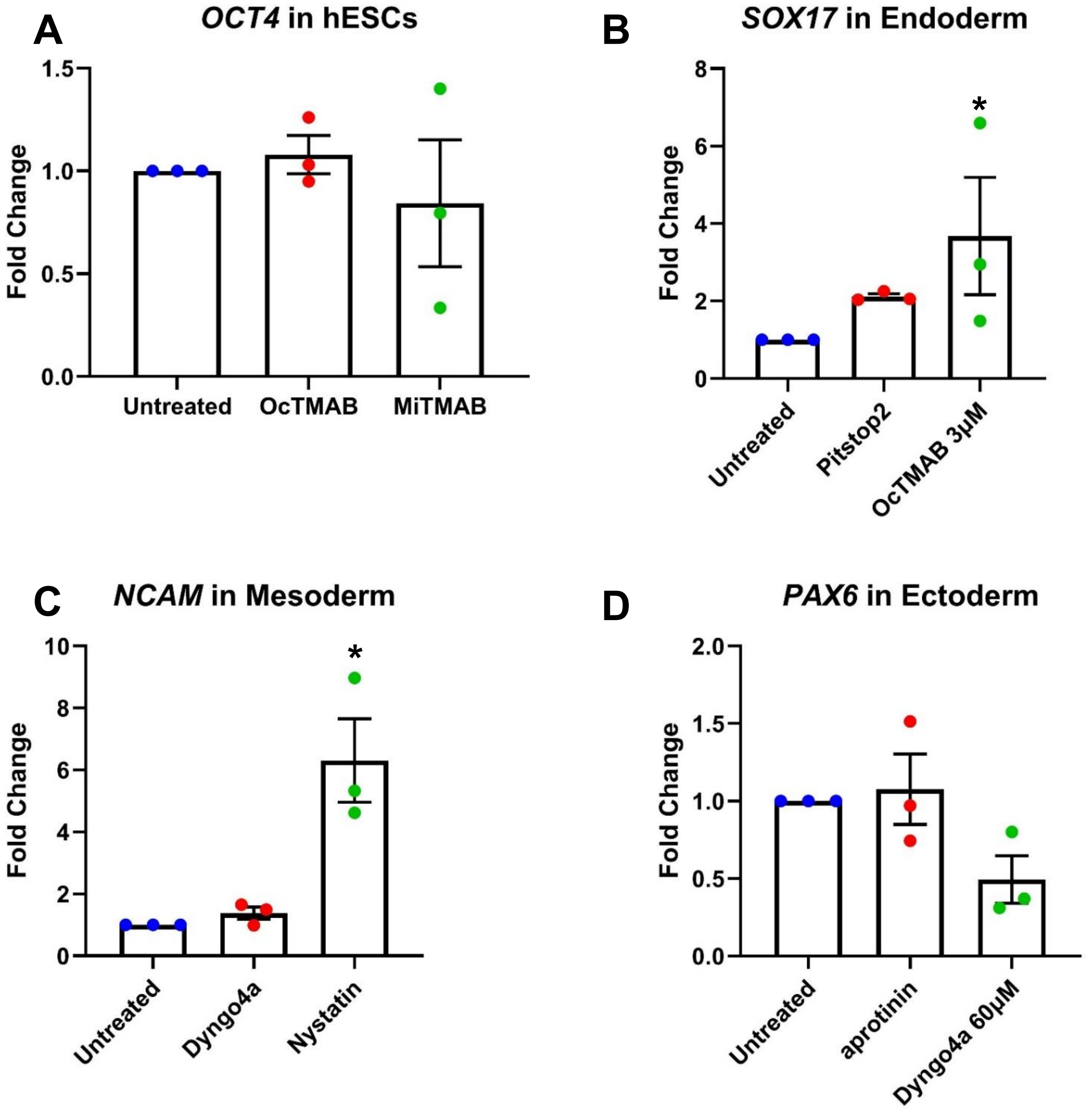

Supplementary Figure 5. Most inhibitors did not significantly alter the expression of the hESC and germ layer markers. qPCR of (A) hESCs, (B) endoderm, (C) mesoderm, and (D) ectoderm was performed in the presence or absence of small molecule inhibitors to confirm the equivalent expression of markers for each cell type in control and treated groups. Kruskal-Wallis non-parametric analyses were performed on the qPCR data. Data are the means ± SEM of three independent experiments. * = $p < 0.05$.

1.2 Supplementary Table

Name	Sequence
OCT4_F	CCTGAAGCAGAAGAGGATCACC
OCT4_R	AAAGCGGCAGATGGTCGTTTGG
PAX6_F	CTGAGGAATCAGAGAAGACAGGC
PAX6_R	ATGGAGCCAGATGTGAAGGAGG
SOX17_F	ACGCTTTCATGGTGTGGGCTAAG
SOX17_R	GTCAGCGCCTTCCACGACTTG
NCAM_F	CATCACCTGGAGGACTTCTACC
NCAM_R	CAGTGTACTGGATGCTCTTCAGG
β-actin_F	CACCATTGGCAATGAGCGGTTC
β-actin_R	AGGTCTTTGCGGATGTCCACGT

Supplementary Table 1. Name and sequences of qPCR primers.

Type of Inhibitor	Name	Mechanism of Action	Tested Cell Lines and Concentrations	DOI for References
TMPRSS2	Aprotinin	Broad-spectrum trypsin inhibitor (Bestle et al., 2020).	Calu-3 (20 µM; Bojková et al., 2020) Human primary epithelial cells (20 µM; Bestle et al., 2020)	Bestle et al., 2020<br>Bojková et al., 2020
	Ambroxol	Predicted to block coronavirus infections by interacting with the active site (glutamine 438) of TMPRSS2 (Shen et al., 2017).	Vero E6 cells (0.1−10 µM; Olaleye et al., 2020)<br>HEK293T, A549 cells (10 µM, no effect; 100-1000 µM; Wang et al., 2023)	Shen et al., 2017<br>Olaleye et al., 2020<br>Wang et al., 2023
	Camostat<br>Nafamostat	Oral serine protease inhibitor, interacts with key residues in the catalytic domain of TMPRSS2 (Hoffmann et al., 2020)	Calu-3 human lung cells (20 µM; Hoffmann et al., 2020, 2021)<br>Human airway epithelial cell (25 µM; Li et al., 2021)<br>Human lung sections (5 µM; Hoffmann et al., 2021)	Hoffmann et al., 2020<br>Hoffmann et al., 2021<br>Li et al., 2021
Endocytosis	Dyngo4a	Blocks dynamin GTPase (Macia et al., 2006)	Human endothelial cells (12.5 µM; Qian et al., 2021)<br>Caco-2 cells (40 µM; Sun et al., 2024)<br>Human renal cells (50 µM) (Somova et al., 2024)<br>HEK293T cells (80 µM) (Bayati et al., 2021)	Macia et al., 2006<br>Qian et al., 2021<br>Sun et al., 2024<br>Somova et al., 2024<br>Bayati et al., 2021
	OcTMAB<br>MiTMAB	Blocks recruitment of dynamin upstream of Dyngo4a (Quan et al., 2007)	Not tested for SARS-CoV-2	Quan et al., 2007
	Pitstop2	Targets the clathrin heavy chain and prevents its interaction with adaptor proteins (Alkafaas et al., 2023).	A549 cells (10 µM; Qu et al., 2024)<br>Human endothelial cells (12.5 µM; Qian et al., 2021)<br>HEK293T cells (15 µM; Bayati et al., 2021)<br>Human renal cells (50 µM; Somova et al., 2024)	Alkafaas et al., 2023<br>Qian et al., 2021<br>Bayati et al., 2021<br>Somova et al., 2024
	Nystatin	Inhibits endocytosis by reducing cholesterol levels (Alkafaas et al., 2023), decreasing caveolae (Dreja et al., 2002).	VeroE6 cells (50 µM, worsened effect; Nguyen et al., 2024)	Alkafaas et al., 2023<br>Dreja et al., 2002<br>Nguyen et al., 2024

Supplementary Table 2. Protease and endocytosis inhibitors. Mechanism of action of small molecule inhibitors targeting SARS-CoV-2 entry and their information on *in vitro* studies are listed in this table.
